# Amygdala vulnerability in Pick disease with discrete frontotemporal dementia syndromes

**DOI:** 10.1111/bpa.70159

**Published:** 2026-10-27

**Authors:** Rachel Keszycki, Allegra Kawles, Grace Minogue, Antonia Zouridakis, Alyssa Macomber, Caroline Nelson, Qinwen Mao, Rudolph J. Castellani, Pouya Jamshidi, Nathan Gill, Sandra Weintraub, M.‐Marsel Mesulam, Changiz Geula, Tamar Gefen

**Affiliations:** ^1^ Mesulam Institute for Cognitive Neurology and Alzheimer's Disease, Feinberg School of Medicine, Northwestern University Chicago Illinois USA; ^2^ Department of Psychiatry and Behavioral Sciences Feinberg School of Medicine, Northwestern University Chicago Illinois USA; ^3^ Department of Pathology Feinberg School of Medicine, Northwestern University Chicago Illinois USA; ^4^ Department of Preventative Medicine Feinberg School of Medicine, Northwestern University Chicago Illinois USA; ^5^ Department of Neurology Feinberg School of Medicine, Northwestern University Chicago Illinois USA; ^6^ Department of Cell and Molecular Biology Feinberg School of Medicine, Northwestern University Chicago Illinois USA; ^7^ Present address: Department of Pathology University of Utah School of Medicine Salt Lake City Utah USA

**Keywords:** amygdala, frontotemporal lobar degeneration, neuroinflammation, neuropsychiatric symptoms, pick disease, tauopathies

## Abstract

Frontotemporal lobar degeneration with tauopathy (FTLD‐tau) typically manifests as either behavioral variant frontotemporal dementia (bvFTD) or primary progressive aphasia (PPA). Tau pathology in FTLD‐tau is characterized by predominantly three‐repeat (3R) tau, as in Pick disease (PiD), or four‐repeat (4R) tau species of corticobasal degeneration (CBD) and progressive supranuclear palsy (PSP). FTLD‐tau preferentially targets frontal and anterior temporal lobes, including the amygdala, contributing to severe neuropsychiatric symptoms. This study investigated whether bvFTD and PPA due to FTLD‐tau exhibit distinct amygdalar histopathology and whether these patterns reflect their initially distinct clinical phenotypes, particularly in PiD. We examined 47 autopsy‐confirmed FTLD‐tau cases from the Northwestern Alzheimer's Disease Research Center Brain Bank using postmortem digital pathology analysis to quantify tau phosphorylation and HLA‐DR immunoreactivity in the amygdala. In a subset of 19 PiD cases (10 bvFTD‐PiD, 9 PPA‐PiD), we assessed neuropsychiatric symptoms, tracked longitudinally using the Neuropsychiatric Inventory–Questionnaire (NPI‐Q), and quantified neuronal and Pick body densities via unbiased stereology and neuronal sizes via digital pathology analysis. Across the FTLD‐tau cohort, bvFTD exhibited greater amygdalar neuroinflammation than PPA, despite comparable tau burden. Interestingly, 3R cases also demonstrated higher levels of phosphorylated tau and MHC‐II‐positive microglia than 4R cases. In PiD, neuropsychiatric symptoms converged over time, with PPA cases evolving to show bvFTD‐like symptoms with disease progression. BvFTD‐PiD showed a trend toward greater MHC‐II‐positive microglia than PPA‐PiD, despite similar neuronal size/density and Pick body burden. Increased HLA‐DR immunoreactivity correlated positively with Pick body pathology. Neurons containing tau pathology (Pick cells) were notably larger than unaffected neurons, suggesting that early tau accumulation precedes overt neuronal degeneration. These findings underscore the potential role of neuroinflammation in shaping clinical phenotypes in FTLD and highlight the role of the amygdala in complex clinicopathologic relationships. Targeting neuroinflammatory processes may represent a viable therapeutic avenue for managing neuropsychiatric symptoms in tauopathies.

## Graphical abstract

Clinicopathological characterization of the amygdala in FTLD‐tau. Amygdala vulnerability in FTLD‐tauopathies is defined by elevated neuroinflammation and tau in 3R versus 4R subtypes, and greater neuroinflammation in bvFTD versus PPA. In Pick disease, bvFTD and PPA converge neuropsychiatrically over time, and intracellular tau correlates with increased neuroinflammation and larger neurons.

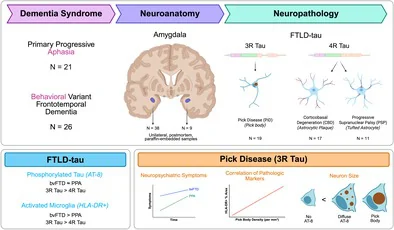

## 1 INTRODUCTION

Frontotemporal lobar degeneration with tauopathy (FTLD‐tau) is a neurodegenerative disease that represents the leading cause of dementia in individuals under 65 [1]. Patients with dementia due to FTLD‐tau commonly present with behavioral variant frontotemporal dementia (bvFTD) with significant behavioral and comportmental dysfunction [2] or primary progressive aphasia (PPA) with salient language dysfunction [3, 4, 5]. Understanding and treating FTLD‐tauopathies is difficult due to heterogeneous relationships between underlying pathology and clinical dementia diagnosis. Namely, a single dementia syndrome can be caused by multiple FTLD‐tau subtypes, including the three‐repeat (3R) tauopathy Pick disease (PiD) and four‐repeat (4R) tauopathies such as corticobasal degeneration (CBD) and progressive supranuclear palsy (PSP). Conversely, patients with the same FTLD‐tau subtype can present with different clinical syndromes. Beyond the frontal and temporal lobes, limbic structures such as the amygdala are also affected in PPA and bvFTD due to FTLD‐tau, causing patients to develop significant neuropsychiatric symptoms.

In this study, we placed particular emphasis on PiD because it shows early, severe, and selective involvement of the basolateral amygdala, whereas amygdala pathology emerges later or more variably in CBD and PSP. Given this disproportionate vulnerability, PiD serves as an ideal model for investigating how region‐specific amygdalar pathology contributes to divergent clinical phenotypes (bvFTD vs. PPA) and the emergence of neuropsychiatric symptoms.

The aims of this study were threefold. First, we sought to determine whether bvFTD and PPA due to FTLD‐tau exhibit distinct patterns of amygdalar pathology corresponding to their initially divergent clinical presentations. Second, we examined the longitudinal progression of neuropsychiatric symptoms in PPA and bvFTD cases with underlying PiD. Finally, we investigated the relationships between neuronal loss and shrinkage, neuroinflammation, and the accumulation of phosphorylated tau and Pick body inclusions to further elucidate how these pathological features may contribute to clinical symptomatology.

## 2 MATERIALS AND METHODS

### 2.1 Case characteristics

Forty‐seven cases (48.93% male) with antemortem clinical diagnoses of bvFTD (*n* = 26) or PPA (*n* = 21) and an autopsy‐confirmed primary pathologic diagnosis of FTLD‐tau who had paraffin‐embedded, unilateral amygdala (38 left, 9 right) tissue available were identified from the NIA‐funded Northwestern Alzheimer's Disease Research Center Brain Bank. Within the total FTLD‐tau cohort, 19 cases had an autopsy‐confirmed primary pathologic diagnosis of PiD (10 bvFTD, 9 PPA), 17 had CBD (11 bvFTD, 6 PPA), and 11 had PSP (5 bvFTD, 6 PPA). Cases with bvFTD were diagnosed using established criteria from the International behavioral variant FTD Consortium [2]. PPA cases were diagnosed according to established criteria [5] with subtypes determined through chart review guided by established standards [6, 7]. Of the 21 PPA cases, 14 (67%) carried the agrammatic subtype as determined through chart review. One case each was diagnosed with the semantic, mixed, and logopenic variants of PPA, and four cases were not subtyped due to disease severity at the time of their initial visit. Seventeen cases (81%) were simultaneously enrolled in the Northwestern PPA program. Written informed consent for brain donation was obtained from all individuals. Table 1 depicts case characteristics.

**TABLE 1:** Case characteristics.

Case	Gender	Education	APOE	CDR global	CDR global	Age at onset	Age at death	Disease duration	Clinical diagnosis	Pathological diagnosis	Amygdala hemisphere	Brain weight	PMI
		(years)		(initial visit)	(final visit)	(years)	(years)	(years)				(grams)	*(hours)*
P1	F	20	3,4	2	3	66	78	12	bvFTD	3R/PiD	Left	1010	25
P2	M	20	2,3	0.5	3	59	67	8	bvFTD	3R/PiD	Right	990	21
P3	F	18	3,3	2	3	70	82	12	bvFTD	3R/PiD	Left	890	9
P4	F	12	2,3	1	3	46	58	12	bvFTD	3R/PiD	Left	866	13
P5	M	12	3,3	0.5	3	59	70	11	bvFTD	3R/PiD	Left	1105	24
P6	F	18	3,3	1	3	58	71	13	bvFTD	3R/PiD	Right	661	2
P7	M	14	NA	0.5	3	60	66	6	bvFTD	3R/PiD	Right	988	7
P8	M	18	NA	2	3	47	61	14	bvFTD	3R/PiD	Left	880	24
P9	M	16	3,3	1	3	62	69	7	bvFTD	3R/PiD	Left	1133	49
P10	M	16	NA	1	2	61	71	10	bvFTD	3R/PiD	Left	1146	27
P11	F	17	3,3	3	3	58	71	13	PPA*	3R/PiD	Left	830	8
P12	F	16	3,4	0.5	3	53	64	11	PPA‐S	3R/PiD	Left	990	28
P13	M	20	3,3	0.5	3	56	70	14	PPA‐G	3R/PiD	Right	1018	5
P14	F	12	3,4	0.5	0.5	63	67	4	PPA‐G	3R/PiD	Right	1140	Unknown
P15	M	12	3,3	0.5	2	62	69	7	PPA*	3R/PiD	Left	1100	5
P16	M	14	NA	0.5	3	66	75	9	PPA*	3R/PiD	Right	1125	27
P17	M	14	3,3	0.5	3	53	65	12	PPA‐G	3R/PiD	Left	1055	Unknown
P18	F	16	3,3	0.5	2	60	68	8	PPA‐G	3R/PiD	Left	1010	14
P19	F	16	3,4	0.5	3	64	76	12	PPA‐G	3R/PiD	Left	965	3.5
P20	M	12	2,3	2	NA	50	54	4	bvFTD	4R/CBD	Left	1320	24
P21	F	14	3,4	2	3	58	62	4	bvFTD	4R/CBD	Left	1040	5
P22	F	14	3,4	1	NA	44	49	5	bvFTD	4R/CBD	Left	1200	16
P23	F	12	3,3	1	3	65	76	11	bvFTD	4R/CBD	Left	910	26
P24	F	14	2,4	0	3	72	81	9	bvFTD	4R/CBD	Left	1030	17
P25	M	20	3,4	2	NA	63	66	3	bvFTD	4R/CBD	Left	1350	7
P26	M	16	NA	0.5	3	60	71	11	bvFTD	4R/CBD	Left	1030	5
P27	M	16	NA	0.5	3	63	67	4	bvFTD	4R/CBD	Right	1226	36
P28	M	16	3,3	0.5	3	62	69	7	bvFTD	4R/CBD	Right	1392	70
P29	M	18	3,4	1	3	65	71	6	bvFTD	4R/CBD	Left	1294	58
P30	F	18	3,3	0.5	3	53	56	3	bvFTD	4R/CBD	Left	1226	24
P31	M	16	3,3	0.5	3	60	69	9	PPA‐G	4R/CBD	Left	1160	18
P32	F	18	3,3	0	3	62	71	9	PPA‐L	4R/CBD	Left	990	9
P33	F	14	3,3	0	3	66	74	8	PPA‐G	4R/CBD	Left	1168	5
P34	F	14	NA	0.5	3	67	72	5	PPA‐G	4R/CBD	Left	980	16
P35	M	20	3,4	0	3	65	83	18	PPA‐G	4R/CBD	Left	1080	8
P36	F	14	3,3	0	3	74	87	13	PPA‐M	4R/CBD	Left	1120	7
P37	M	18	2,3	1	1	71	77	6	bvFTD	4R/PSP	Left	1240	8
P38	F	16	3,3	1	2	67	72	4	bvFTD	4R/PSP	Left	1250	49
P39	M	15	3,3	1	2	67	75	8	bvFTD	4R/PSP	Left	1170	12
P40	M	16	3,3	1	3	75	83	8	bvFTD	4R/PSP	Left	1080	20
P41	M	16	3,3	2	NA	62	71	9	bvFTD	4R/PSP	Left	1250	Unknown
P42	F	16	NA	3	NA	73	82	9	PPA‐G	4R/PSP	Left	1170	Unknown
P43	M	20	2,3	0.5	1	80	87	8	PPA‐G	4R/PSP	Left	1315	7
P44	F	14	3,4	0	0.5	77	82	5	PPA*	4R/PSP	Left	1280	16
P45	F	16	3,3	0	3	70	82	12	PPA‐G	4R/PSP	Left	1060	9
P46	F	16	3,3	0	0	73	84	11	PPA‐G	4R/PSP	Left	1250	67
P47	F	14	3,3	0	3	78	88	10	PPA‐G	4R/PSP	Right	970	7

Abbreviations: CDR, Clinician Dementia Rating Scale; F, female; M, male; P1‐47, participants with dementia due to FTLD‐tau; PMI, postmortem interval; PPA‐S, primary progressive aphasia, semantic variant; PPA‐G, primary progressive aphasia, agrammatic variant; PPA‐L, primary progressive aphasia, logopenic variant; PPA*, primary progressive aphasia, not subtyped (too severe); 3R/PiD, three‐repeat tauopathy/Pick disease; 4R/CBD, four‐repeat tauopathy/corticobasal degeneration; 4R/PSP, four‐repeat tauopathy/progressive supranuclear palsy.

### 2.2 Neuropsychiatric symptom assessment

At each research visit, neuropsychiatric symptoms were quantified for all cases via the Neuropsychiatric Symptom Inventory–Questionnaire, a 12‐item, informant‐based questionnaire that assesses the presence or absence of common neuropsychiatric symptoms in dementia [8]. In PPA‐PiD and bvFTD‐PiD cases specifically, we examined whether clinical groups differed in the level of behavioral / comportmental symptoms (i.e., apathy, disinhibition, motor stereotypies, and appetite disturbance) [9] endorsed at their initial and final research visits.

### 2.3 Neuropathologic assessment and histological preparation

The cerebral hemispheres were divided along the midsagittal plane, sliced into 2–3‐cm coronal blocks, and preserved in formalin for 2 weeks or 4% paraformaldehyde for 36 h. They were subsequently taken through sucrose gradients (ranging from 10% to 40%) for cryoprotection and stored in a solution of 40% sucrose with 0.02% sodium azide at 4°C. For neuropathological diagnosis, small tissue samples were embedded in paraffin and stained with H&E or Thioflavin‐S (Sigma‐Aldrich) or immunohistochemically stained to visualize phosphorylated tau (AT‐8), p62 (BD Biosciences), phosphorylated TDP‐43 (pS409‐410‐2, Cosmo Bio), phosphorylated alpha‐synuclein (pS129), and beta‐amyloid (4G8, Signet). The neuropathological diagnosis of FTLD and identification of its subtypes were confirmed by neuropathologists (EB, QM, RJC) using the established consensus criteria of the Consortium for FTLD [10].

All cases showed an FTLD‐tauopathy (3R/PiD, 4R/CBD, or 4R/PSP) as the primary pathologic diagnosis with no evidence of other primary tauopathies. Additional neuropathological assessment of 3R tau (RD3 (clone 8E6/C11); mouse monoclonal; Sigma‐Aldrich; 1/1000) and 4R tau (ET3; gift of Dr. Peter Davies; 1/1000) revealed tau inclusions were predominantly comprised of the 3R tau isoform in 3R/PiD cases and of the 4R tau isoform in 4R/CBD and 4R/PSP cases (Supplementary Figure 1). No cases met criteria for FTLD due to TDP‐43 neuropathology; 2 cases with PPA due to PSP pathology showed mild medial temporal TDP‐43 pathology [11]. Cases did not carry any known mutations linked to AD or FTLD. No cases had higher than “intermediate” Alzheimer's Disease neuropathologic change (ADNC), with all cases at Braak stage III or lower [12]. Cases with comorbid amygdala‐predominant or neocortical Lewy body disease [13] were excluded (*n* = 2 PSP and *n* = 1 CBD). No cases had aging‐related tau astrogliopathy (ARTAG) [14]. The presence of comorbid argyrophilic grain disease (AGD) was permitted [15].

For all cases, samples were taken from unilateral amygdala, including 38 from the left hemisphere (17 PPA and 21 bvFTD) and 9 from the right hemisphere (4 PPA and 5 bvFTD). Amygdala samples were embedded in paraffin and cut into 5 μm‐thick sections, which were stained to visualize neurons, phosphorylated tau, or MHC‐II‐positive microglia. For neurons, sections were histologically stained using 1.0% Cresyl violet. To visualize phosphorylated tau, sections were stained immunohistochemically with the AT‐8 antibody, which recognizes human tau phosphorylated at Ser202/205 (mouse monoclonal; Invitrogen MN1020; 1/500). MHC‐II‐positive microglia were detected via immunohistochemical staining using an antibody against the human leukocyte antigen‐d related protein (HLA‐DR, mouse monoclonal; Invitrogen MA125914; 1/100). AT‐8 and HLA‐DR staining used the Avidin–Biotin Complex (ABC)‐based method with streptavidin and were developed manually using amino ethyl carbazol (AEC) chromogen (NovaRed; Vector Laboratories SK4800).

Stained amygdala sections were contoured for analysis with guidance from a neuroanatomist (CG) and a coronal human brain atlas [16]. Briefly, the amygdala was defined by neuroanatomical landmarks including the anterior aspect of the hippocampal formation and anterior edge of the inferior horn of the lateral ventricle, below the uncus. We quantified histopathological markers and neurons within the basolateral region, comprised of the lateral, basal, and accessory basal nuclei, chosen based on their extensive frontotemporal connections [17].

### 2.4 Digital quantification of AT‐8 and HLA‐DR staining

The Olympus V200 slide scanner was used to obtain digital images of slides at 20× magnification. Images of amygdala were manually annotated using HALO digital image software v3.5.3 (Indica Labs). Then, percent area occupied (%AO), or the total percentage area covered by AT‐8 or HLA‐DR immunoreactivity, was obtained using the Object Colocalization algorithm. In line with prior methods [18], the optimal color and minimum tissue optical density were averaged among a subset (i.e., 20%) of the total slides to optimize analysis parameters for all slides. Cases with %AO values outside the mean ± 1 SD underwent manual quality control for accuracy, and values were adjusted accordingly.

### 2.5 Modified stereological analysis of Pick body and neuronal density

Modified stereological analysis was carried out according to procedures previously described in detail [19, 20], employing the fractionator method and StereoInvestigator software (MicroBrightField) to estimate the density of Pick bodies and neurons. Five adjacent 5‐μm paraffin sections through the amygdala were sampled per case. For each section, the amygdala was traced at 2.5× magnification, and systematic‐random sampling was applied using counting frames of 100 × 100 μm. The top and bottom 1 μm of each section were designated as guard zones, yielding an optical dissector height of 3.0 μm per slide and total sampling depth of 15 μm across 5 slides per case. Pick bodies and neurons were counted if they came into focus within the *i*‐axis of the chosen dissector height of 3.0 μm. Regions were analyzed at 40× for neurons and 63× for Pick bodies to ensure Pick bodies were clearly visualized and consistently distinguished from background staining and other tau‐positive structures. All parameters were adjusted to keep the coefficient of error <0.1. Stereological counts from all sampled sections were expressed as mean counts per mm^3^ based on planimetric calculation of volume by the fractionator software. Mean densities were compared between clinical groups, and Pick‐body‐to‐neuron ratios were computed to analyze the relationship between inclusion burden and neuronal density.

### 2.6 Digital neuronal sizing

Slides stained for Cresyl violet (CV) were digitally imaged at 20× magnification using an Olympus V200 slide scanner system (Olympus, Tokyo, Japan) then uploaded into QuPath v.0.6.0, an open‐source software used for Quantitative Pathology and Bioimage Analysis [21]. Sections stained with CV were first manually contoured using the polygon tool. Then, 2.5% of the total contour area was delineated by 250 × 250 μm boxes, which were added randomly inside the contour using a customized QuPath script (see https://github.com/keszy001/QuPath-random-ROI-generator). All neurons were then manually sized in μm^2^ within each box using the polygon tool. Manual tracing was performed in accordance with our previously published methods showing sufficient inter‐rater reliability [22]. Convergence analysis in 3 cases (bvFTD: P2 and P3; PPA: P17) indicated stable neuronal size means and standard deviations for each case at our chosen sampling fraction of 2.5% (see Supplementary Figure 2). As Pick bodies are an intraneuronal pathology, we double‐stained 5 PiD cases (3 bvFTD cases: P1, P5, and P9 and 2 PPA cases: P16 and P19) with AT‐8 and CV and separately sized neurons with Pick body inclusions, neurons with diffuse AT‐8 staining, and intact neurons devoid of AT‐8 immunoreactivity based on previously published works identifying pre‐inclusions versus mature inclusions [23, 24]. Neurons containing diffuse phosphorylated tau or Pick bodies were of similar size, both of which were significantly larger than neurons devoid of AT‐8 immunoreactivity on average (Figure 1A–C). Therefore, when sizing CV‐stained neurons, we separately sized intact neurons and “Pick cells,” defined as neurons with swollen cytoplasm, presumably bearing tau, based on previously well‐described morphology [25, 26, 27]. Mean total area of intact neurons, Pick cells, and their average was calculated across three CV‐stained amygdala sections per case and evaluated for differences between clinical groups (PPA vs. bvFTD).

**FIGURE 1:**
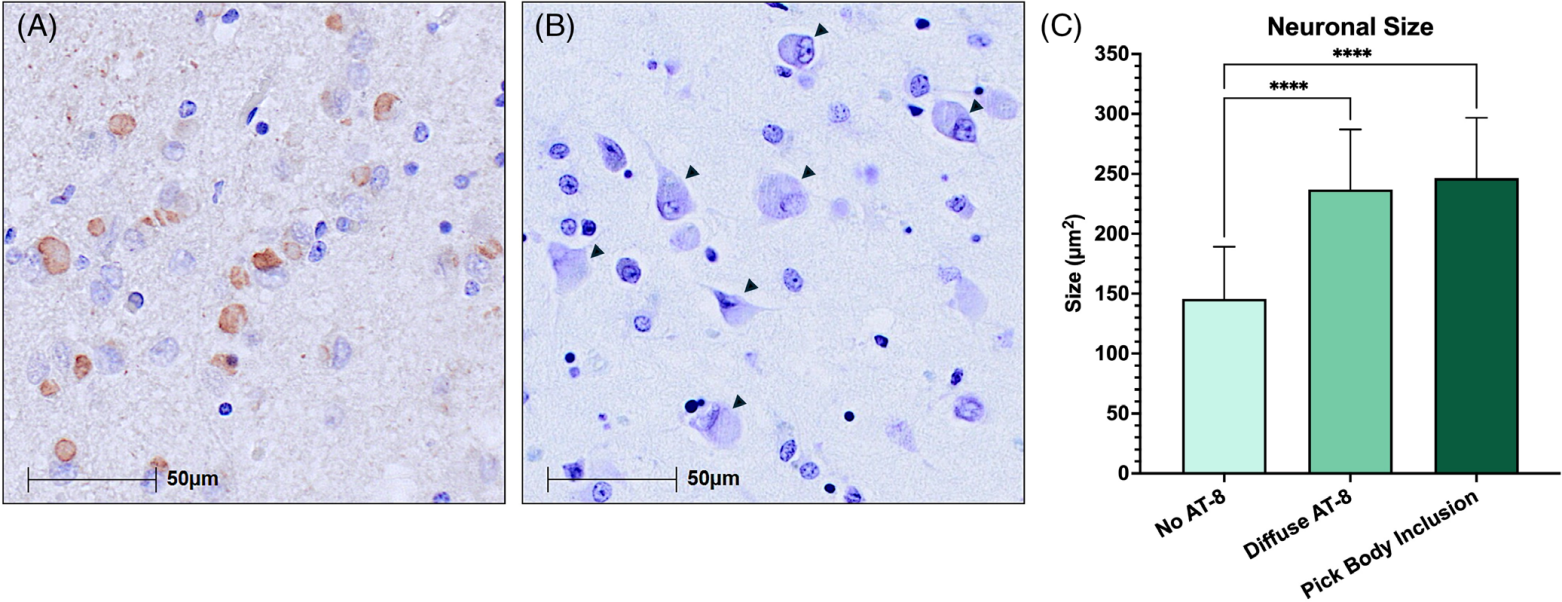
Neuronal size in Pick disease at different stages of tau accumulation. Photomicrographs of bvFTD‐PiD case P1 amygdala taken at 20×show considerable variability in neuronal size, both in neurons double‐stained for AT‐8 and CV (A) and in CV‐stained neurons (B) (see Table 1). In (A), phosphorylated tau‐bearing neurons in this Pick disease case are characterized by intraneuronal Pick bodies and diffusely aggregated tau in the cytoplasm. CV staining (B) shows abundant Pick cells, indicated by black arrowheads, which are characterized by ballooned cytoplasm and a displaced nucleus. As indicated in (C), neurons bearing diffuse phosphorylated tau or Pick body inclusions are significantly larger than intact neurons devoid of AT‐8 immunopositivity (both *p*< 0.0001, *N* = 5 cases: P1, P5, and P9 with bvFTD and P16 and P19 with PPA). Graph depicts means ± SEM.

### 2.7 Statistical analysis

Across the FTLD‐tau cohort, we fit separate two‐way ANOVA models to test whether age at symptom onset, age at death, disease duration, brain weight, and postmortem interval (PMI) differed by clinical diagnosis (bvFTD vs. PPA), FTLD‐tau species (3R vs. 4R), and their interaction. We conducted Fisher's exact test to determine whether APOE allele frequencies differed between clinical diagnosis or FTLD‐tau species. Missing values were handled via listwise deletion. To test whether clinical diagnosis and tau species predicted HLA‐DR or AT‐8 percent immunopositivity in the amygdala, we fit multivariable linear regression models with hemisphere, disease duration, and PMI included as covariates. Hemisphere was included based on our prior studies showing asymmetric disease burden in PPA [19, 28]. Disease duration was included given prior associations with glial activation and phosphorylated tau burden in tauopathies [29]. PMI was included given that HLA‐DR immunoreactivity may be sensitive to postmortem delay [30]. As secondary analyses, we tested whether HLA‐DR or AT‐8 immunopositivity differed by the presence/absence of comorbid ADNC or AGD using Welch's *t*‐tests.

In the PiD subset, demographic variables were compared between clinical groups using Welch's *t*‐tests. Differences in behavioral/comportmental NPI‐Q symptoms between clinical groups at initial and final visits were assessed using mixed‐effects linear models including clinical diagnosis, timepoint, and their interaction as fixed effects, along with subject ID as a random intercept to account for repeated measures, followed by planned contrasts. Differences in pathological markers between clinical groups in the PiD subset were additionally evaluated using multivariable linear regression models including hemisphere and disease duration as covariates. Pearson correlations assessed relationships among pathological variables. In 5 cases from the PiD subset double‐stained with CV and AT‐8 to visualize neurons and phosphorylated tau, we fit a mixed‐effects linear model to assess for the difference in neuronal size between neurons without AT‐8 immunopositivity, neurons with diffuse AT‐8 immunopositivity, and neurons bearing Pick bodies, including subject ID as a random intercept.

All statistical analyses were performed in RStudio version 2026.07.1 + 147 (R version 4.4.1; Posit Software, Boston, MA, USA; see https://github.com/keszy001/StatsFTLDTauAll_2026 for R code). Significance level was set to *p*< 0.05. Effect sizes were quantified using partial omega‐squared (ω^2^_p_). When warranted, we performed post hoc pairwise comparisons of estimated marginal means for ANOVA and regression models with Benjamini–Hochberg false discovery rate correction.

## 3 RESULTS

### 3.1 Total FTLD‐tau cohort

#### 3.1.1 Demographics

BvFTD cases were characterized by a relatively younger average age at onset than PPA cases (bvFTD *M* = 60.96 years, PPA *M* = 65.71 years, *p*< 0.05). Two‐way ANOVA indicated a significant interaction between clinical diagnosis (bvFTD, PPA) and FTLD‐tau species (3R, 4R) for disease duration (*p*< 0.05), which was shortest for bvFTD cases with 4R FTLD‐tau pathology (*M* = 6.38 years), significantly shorter than bvFTD with 4R tau, PPA with 3R tau, and bvFTD with 3R tau (all *p*< 0.05). Collapsing across tau species, bvFTD cases were characterized by a relatively shorter mean disease duration than PPA cases (bvFTD *M* = 7.96 years, PPA *M* = 9.86 years, trend, *p* = 0.05). Two‐way ANOVA also indicated a significant interaction between clinical diagnosis and FTLD‐tau species for average age at death (*p*< 0.05), which was oldest for PPA with underlying 4R FTLD‐tau (*M* = 80.08 years), significantly older than all other clinical‐by‐tau‐species groups (all *p*< 0.01). Collapsing across tau species, bvFTD cases were characterized by a relatively younger average age at death than PPA cases (bvFTD *M* = 68.96 years, PPA *M* = 75.52 years, *p*< 0.01). Clinical groups did not significantly differ by brain weight (bvFTD *M* = 1102.96 grams, PPA *M* = 1084.57 grams) or PMI (bvFTD *M* = 23.12 h, PPA *M* = 14.42 h). Irrespective of clinical diagnosis, 3R and 4R tau groups differed notably in all demographic variables besides average PMI (3R *M* = 17.15 h, 4R *M* = 21 h). Relative to 4R tau cases, 3R tau cases were characterized by younger age at symptom onset (3R *M* = 59.11 years, 4R *M* = 65.79 years, *p*< 0.01) and death (3R *M* = 69.37, 4R *M* = 73.61 years, *p*< 0.05), longer disease duration (3R *M* = 10.26 years, 4R *M* = 7.82 years, *p*< 0.05), and lower brain weight (3R *M* = 994.84 grams, 4R *M* = 1162.54 grams, *p*< 0.001).

Apolipoprotein E (ApoE) genotyping data was available for 39 cases with the following allele frequencies: 7.69% ε2, 78.21% ε3, and 14.10% ε4. There were no statistically significant variances in ApoE ε4 frequency between clinical or tauopathy groups. Comorbid “low” or “intermediate” ADNC [12] was present in approximately half of the total FTLD‐tau cohort, including 56% of 3R FTLD‐tau cases (*n* = 10) and 50% of 4R FTLD‐tau cases (*n* = 14). Consistent with prior reports [15], comorbid AGD was present in 43% of 4R FTLD‐tau cases (*n* = 12) but none of the 3R FTLD‐tau cases. In the full FTLD‐tau cohort, HLA‐DR and AT‐8 percent immunopositivity did not significantly differ based on the presence or absence of comorbid ADNC. In the 4R FTLD‐tau group specifically, the presence or absence of comorbid AGD was also not associated with HLA‐DR nor AT‐8 percent immunopositivity.

#### 3.1.2 BvFTD cases with 3R/PiD show the highest burden of amygdala pathology

Irrespective of underlying FTLD‐tau species, clinical groups did not significantly differ in average percent area covered by AT‐8 staining for phosphorylated tau (bvFTD *M* = 4.67%, PPA *M* = 6.05%) (Figure 2A,E–L). The bvFTD group had a higher average level of percent HLA‐DR immunopositivity (*M* = 1.81%) than the PPA group (*M* = 0.95%) (ω^2^_p_ = 0.09, 90% CI [0.00, 1.00]; estimated mean difference = 1.43%, 95% CI [−0.04, 2.90]; *p*< 0.05) (Figure 2B,E–L). Interestingly, regardless of clinical diagnosis, cases with an underlying 3R tauopathy (PiD) showed significantly more pathology in the amygdala overall than those with an underlying 4R tauopathy (CBD or PSP). Average burden of phosphorylated tau was higher in the 3R group (*M* = 9.09%) than the 4R group (*M* = 2.70%) (ω^2^_p_ = 0.10, 90% CI [0.01, 1.00]; estimated mean difference = 5.18%, 95% CI [0.80, 9.56]; *p*< 0.05) (Figure 2C, E–L). Percent area covered by HLA‐DR immunoreactivity was also higher in 3R (*M* = 2.57%) than in 4R cases (*M* = 0.67%) (ω^2^_p_ = 0.13, 90% CI [0.02, 1.00]; estimated mean difference = 1.79%, 95% CI [0.26, 3.32]; *p*< 0.05) (Figure 2D,E–L).

**FIGURE 2:**
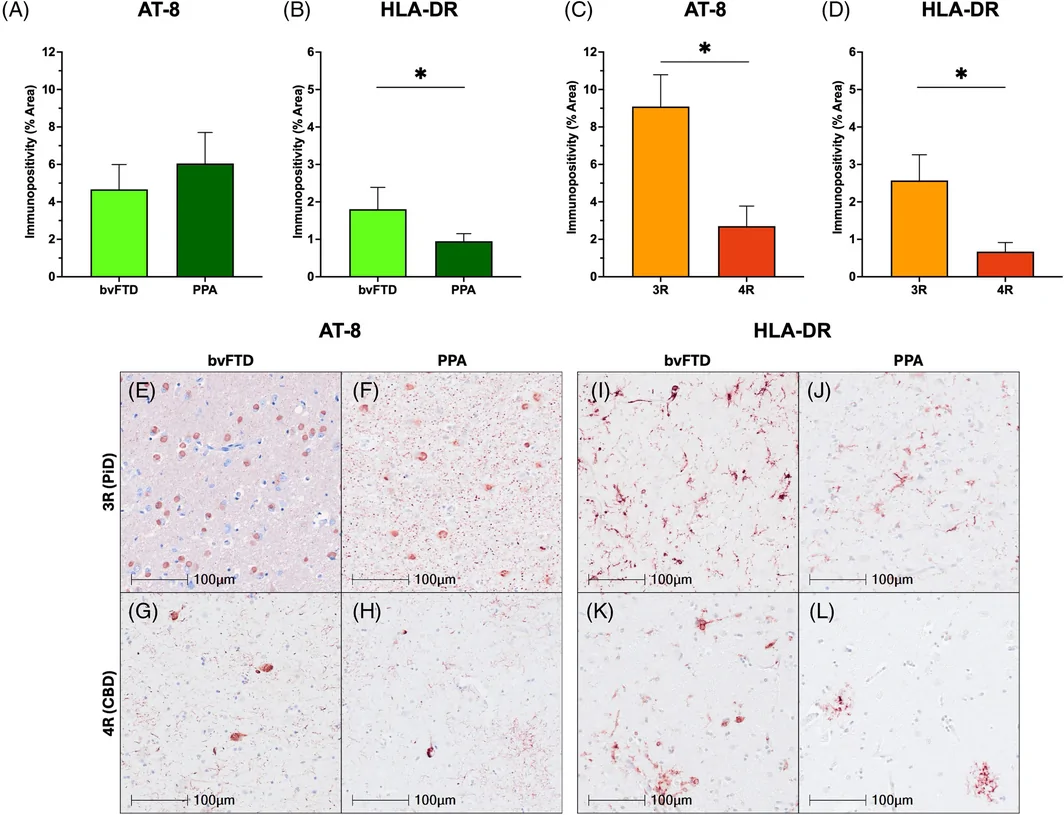
Phosphorylated tau and MHC‐II‐positive microglia in bvFTD and PPA due to 3R and 4R FTLD‐tau. (A) Percent area of amygdala occupied by phosphorylated tau (AT‐8) did not significantly differ between bvFTD and PPA. (B) BvFTD showed more HLA‐DR immunoreactivity than PPA (HLA‐DR percent area, *p*< 0.05). (C) 3R tau showed significantly more phosphorylated tau pathology than 4R tau (*p*< 0.05). (D) Percent area of HLA‐DR immunopositivity was significantly higher for 3R versus 4R tau (*p*< 0.05). Graphs (A)–(D) depict means ± SEM. (E)–(L): 10× Photomicrographs of amygdala immunostained with AT‐8 (E)–(H) and HLA‐DR (I)–(L) in P2: BvFTD‐PiD (E), (I), P18: PPA‐PiD (F), (J), P30: BvFTD‐CBD (G), (K), and P34: PPA‐CBD (H), (L) (see Table 1). *p< 0.05.

### 3.2 PiD cases

#### 3.2.1 Demographics

Clinical groups did not significantly differ in age at onset, age at death, disease duration, brain weight, or PMI. The mean age of symptom onset was 58.80 years (SD = 7.44) for bvFTD and 59.44 years (SD = 4.75) for PPA. Across all PiD cases, duration of illness ranged from 4 to 14 years (*M* = 10.26 years, SD = 2.88), with one PPA case (P14) dying of an acute cause resulting in a notably shorter disease duration. On average, age at death was 69.30 years (SD = 7.12) for bvFTD and 69.44 years (SD = 4.10) for PPA. Overall, the average PMI was 17.15 h (SD = 12.47), and the mean brain weight was 994.84 grams (SD = 126.52). See Table 1 for individual case characteristics.

#### 3.2.2 Convergence in neuropsychiatric symptoms over time

Mixed‐effects models showed significant main effects of clinical diagnosis (ω^2^_p_ = 0.25, 90% CI [0.05, 1.00]; *p*< 0.05) and timepoint (ω^2^_p_ = 0.33, 90% CI [0.10, 1.00]; *p*< 0.01) on behavioral/comportmental NPI‐Q symptoms. Planned contrasts indicated that at their initial research visits, bvFTD‐PiD cases showed more behavioral/comportmental on average than the PPA‐PiD group (bvFTD‐PiD *M* = 2.10 vs. PPA‐PiD *M* = 0.63; estimated mean difference = 1.51, 95% CI [0.48, 2.54], *p*< 0.05), whereas clinical groups did not significantly differ at their final research visits (bvFTD‐PiD *M* = 2.60 vs. PPA‐PiD M = 2.00) (Figure 3). Within‐group contrasts showed that symptoms increased significantly over time in the PPA‐PiD group (initial *M* = 0.63 vs. final *M* = 2.00, estimated mean difference = 1.41, 95% CI [0.46, 2.35], *p*< 0.05), while the bvFTD‐PiD group showed a non‐significant increase (initial *M* = 2.10 vs. final *M* = 2.60). Together, these results suggest convergence in neuropsychiatric presentations between clinical groups over time.

**FIGURE 3:**
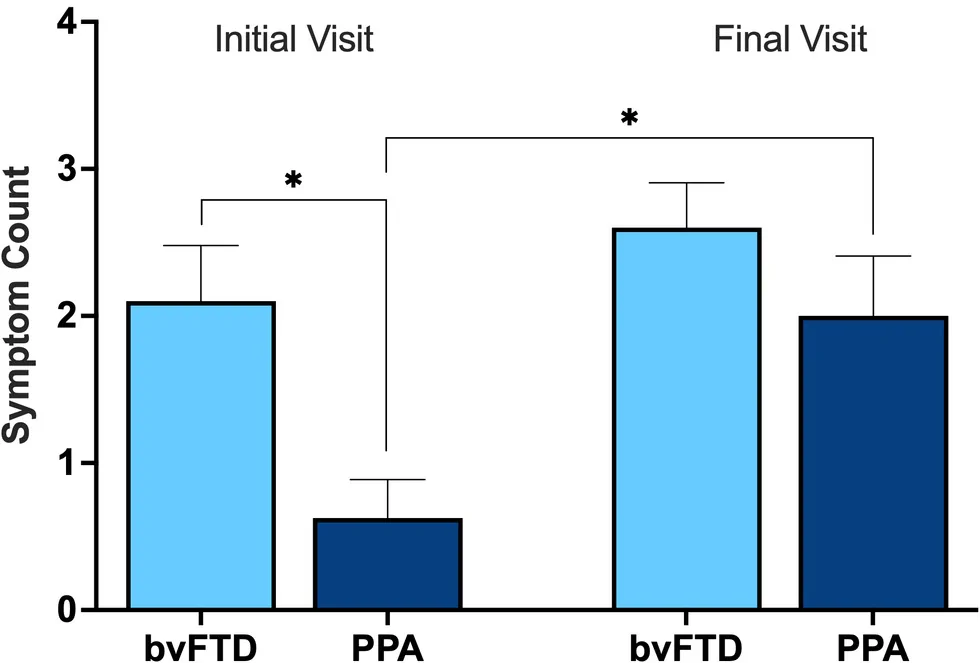
Behavioral/comportmental symptoms in bvFTD and PPA due to Pick disease. Bars represent means ± SEM NPI‐Q symptom counts of apathy, disinhibition, motor stereotypies, and appetite disturbance. At initial research visits, bvFTD‐PiD participants showed significantly more symptoms than PPA‐PiD participants (bvFTD *M* = 2.1 vs. PPA *M* = 0.63, *p*< 0.05). Symptom levels were similar between groups at final visits, with a significant increase over time in the PPA‐PiD group (initial *M* = 0.63, final *M* = 2.0, *p*< 0.05). *p< 0.05.

#### 3.2.3 Higher neuroinflammation in the bvFTD vs PPA phenotype

In PiD cases specifically, bvFTD cases showed a trend toward higher mean percent area of HLA‐DR immunopositivity (*M* = 3.80%) than PPA (*M* = 1.34%) with a moderate effect size (ω^2^_p_ = 0.11, 90% CI [0.00, 1.00]; estimated mean difference = 2.47%, 95% CI [−0.44, 5.37]), though this did not reach statistical significance (*p* = 0.09) (Figure 4I–K). Clinical groups did not notably differ in average neuronal density (bvFTD *M* = 124,733.59/mm^3^, PPA *M* = 142,023/mm^3^), neuronal size (bvFTD *M* = 138.54 μm^2^, PPA *M* = 157.16 μm^2^) (Figure 4A–D), Pick body density (bvFTD *M* = 29,870.05/mm^3^, PPA *M* = 25,113.93/mm^3^), or the Pick‐to‐neuron ratio (bvFTD *M* = 0.28, PPA *M* = 0.18) (Figure 4E–H).

**FIGURE 4:**
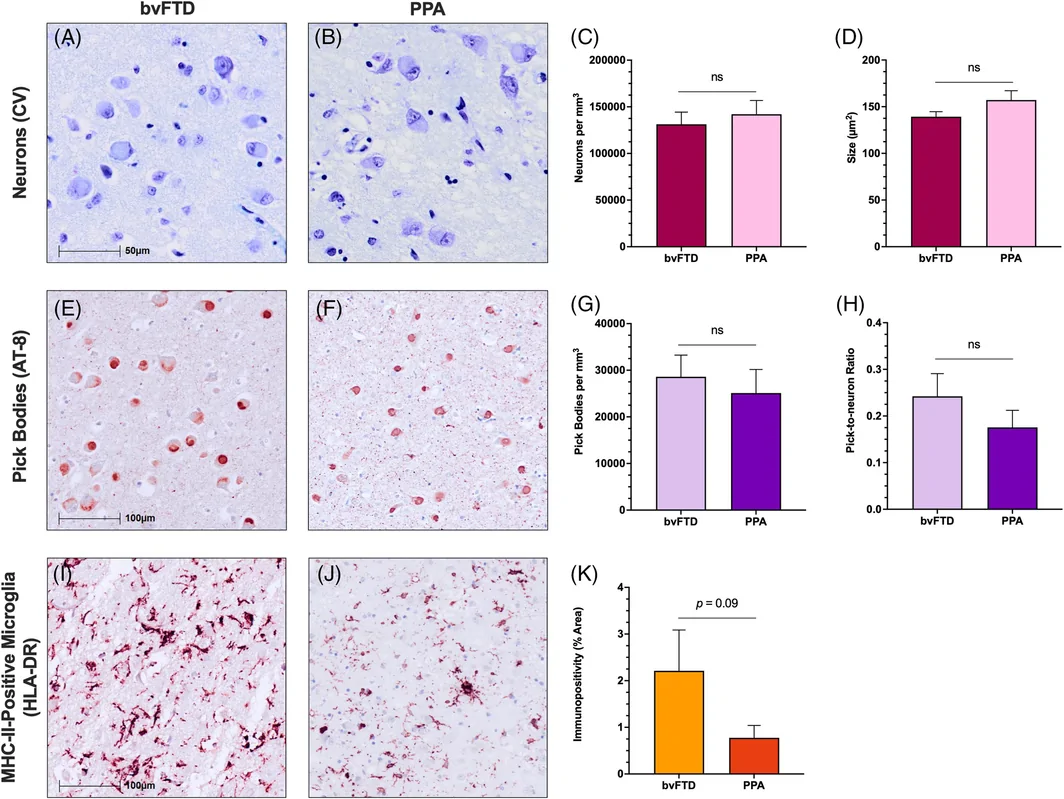
Histopathological markers in bvFTD and PPA due to Pick disease. (A)–(D) Neuronal density per mm^3^ and average neuronal size were similar between bvFTD‐PiD and PPA‐PiD. Photomicrographs taken at 20× magnification depict CV‐stained neurons in bvFTD case P4 and PPA case P18. (E)–(H) Pick body density per mm^3^ and ratio of Pick bodies to neurons were also similar between bvFTD‐PiD and PPA‐PiD. Photomicrographs taken at 10× magnification depict AT‐8‐stained Pick body inclusions in bvFTD case P10 and PPA case P14 (see Table 1). (I)–(K) Level of HLA‐DR immunopositivity was relatively higher in bvFTD‐PiD than in PPA‐PiD (*p* = 0.09). Photomicrographs taken at 10× magnification show HLA‐DR‐stained MHC‐II‐positive microglia in bvFTD‐PiD case P2 and PPA‐PiD case P19. Graphs (C), (D), (G), (H), and (K) depict group means ± SEM.

#### 3.2.4 Association between MHC‐II‐positive microglia and intraneuronal Pick body inclusions

Across all PiD cases, HLA‐DR percent area of immunopositivity was positively correlated with Pick body density (Pearson *r* = 0.54, *r* ^2^ = 0.29, *p*< 0.05), suggesting greater MHC‐II‐positive microglia in cases with higher Pick body burden. This relationship remained significant when controlling for neuronal density (Pearson partial *r* = 0.54, partial *r* ^2^ = 0.29, *p*< 0.05). HLA‐DR percent area was not significantly correlated with neuronal density or measures of neuronal size.

#### 3.2.5 Larger neurons in the presence of greater tau pathology

In accordance with characteristic histopathological features [23, 25, 26, 27, 31], phosphorylated tau aggregated in the cytoplasm of neurons bearing pre‐inclusions or round, well‐defined, Pick bodies, often displacing the nucleus from the midline (Figure 1A). On CV‐stained slides, frequent Pick cells were noted with enlarged, ballooned, vacuolated cytoplasm, typically lacking Nissl substance (Figure 1B). Analyses of neurons double‐stained for AT‐8 and CV in 5 cases (3 with bvFTD: P1, P5, and P9 and 2 with PPA: P16 and P19) revealed a significant effect of neuron type (ω^2^_p_ = 0.92, 90% CI [0.81, 1.00]; *p*< 0.0001). Specifically, we observed a similar mean size between neurons bearing Pick bodies (*M* = 246.47 μm^2^) or diffuse AT‐8 (*M* = 236.97 μm^2^) immunoreactivity, which were both significantly larger than the mean size of neurons without AT‐8 immunoreactivity (*M* = 145.70 μm^2^) (estimated mean difference of empty vs. diffuse AT‐8 neurons = −91.3 μm^2^, 95% CI [−121.50, −61.00]; estimated mean difference of empty vs. Pick body neurons = −100.8 μm^2^, 95% CI [−131.00, −70.50]; both *p*< 0.0001) (Figure 1C). The average size of neuronal soma in intact neurons was positively associated with the density of Pick bodies (Pearson *r* = 0.49, r^2^ = 0.29, *p*< 0.05).

## 4 DISCUSSION

This study characterized postmortem neuropathological changes in the amygdala that distinguish bvFTD and PPA due to 3R and 4R FLTD‐tauopathies. Given the pronounced amygdala pathologic burden of 3R cases, our investigation then focused on characterizing the longitudinal progression of neuropsychiatric symptoms in bvFTD‐PiD versus PPA‐PiD, relationships between histopathologic markers, and how pathologic changes may contribute to clinical symptomatology in PiD. Analyses led to 5 main findings: (1) pathologic burden was more severe in 3R than 4R cases, (2) bvFTD cases showed higher inflammation than PPA cases despite similar levels of other pathologic changes, (3) neuroinflammation correlated with a higher Pick body density across all PiD cases, (4) cases with bvFTD‐PiD and PPA‐PiD showed convergence in neuropsychiatric symptoms over time, and (5) Pick cells were larger than intact neurons in PiD cases.

The amygdala was more severely impacted in 3R compared to 4R tau, as reflected by elevated phosphorylated tau and MHC‐II‐positive microglia. While direct postmortem comparisons of amygdalar pathology across FTLD‐tau subtypes are limited, our results complement prior MRI‐based work [17] showing more pronounced amygdala involvement in 3R than in 4R FTLD‐tauopathies. This pattern likely reflects fundamental differences in neurobiological mechanisms of spread; for instance, tau deposition appears to target the amygdala early in PiD [23], whereas amygdalar involvement emerges later in the 4R FTLD‐tauopathies [32, 33]. Consistent with our results, findings of prior studies also suggest more pronounced neuroinflammation in PiD compared to other tauopathies [34, 35]. This neuroinflammatory response could, in theory, play a role in propagating tau pathology and further neurodegeneration [36, 37], although more research is needed to determine the underlying mechanisms of this differential vulnerability.

Overall, bvFTD cases exhibited significantly greater amygdalar neuroinflammation compared to PPA across the broader FTLD‐tau cohort, with a similar trend in PiD cases specifically, despite similar levels of total phosphorylated tau burden (in FTLD‐tau), Pick body density (in PiD), and neuronal loss and shrinkage (in PiD). Despite bvFTD‐PiD and PPA‐PiD not showing statistically significant differences in MHC‐II‐positive microglia or Pick body density, PiD cases with a higher Pick body density also tended to be those with higher levels of MHC‐II‐positive microglia, even when covarying for neuronal density, although these correlative patterns should be interpreted cautiously given the small sample size. These nuanced relationships between tau burden and neuroinflammation suggest that factors beyond local tau pathology, such as crosstalk with astrocytes and oligodendrocytes as well as white matter degeneration and functional connectivity changes, may influence regional inflammatory responses and clinical symptoms. Indeed, microglia have been shown to interact with astrocytes and oligodendrocytes, both in normal development and aging and in pathological states, including tauopathies [38, 39]. This raises questions about how reactive astrogliosis and oligodendroglial dynamics influence tau‐microglial relationships and suggests that future studies investigating additional glial‐specific markers are warranted. Further, microglial activation has been shown to be more pronounced in white matter relative to adjacent gray matter [40, 41]. BvFTD is characterized by more severe disruption of frontotemporal white matter tracts than agrammatic PPA—the predominant PPA subtype in our cohort [42]. Therefore, pronounced degeneration of tracts connecting the amygdala to frontotemporal regions [42, 43, 44] could result in an elevated presence of MHC‐II‐positive microglia in the amygdala in bvFTD. In the absence of significantly more neuronal shrinkage or death in the amygdala, intrinsically altered activity in the amygdala and altered functional connectivity between to fronto‐parietal regions may also be associated with salient behavioral and neuropsychiatric changes, as has been shown in other neurodegenerative diseases [45]. Future studies of network‐level degeneration and immune signaling are likely to reveal valuable insights into regional vulnerability and clinical expression in FTLD‐tauopathies.

In line with clinical diagnostic criteria [2] and prior findings from our group [9], bvFTD cases were characterized by a higher degree of behavioral/comportmental symptoms, particularly more frequent apathy and motor stereotypies. Notably, however, PPA cases became neuropsychiatrically more “bvFTD‐like” with disease progression, suggesting convergence in clinical presentations over time. This indicates that, despite originating in distinct regions associated with divergent clinical dementia syndromes, Pick's disease likely has a predilection for certain regions [19] and cell types [20], causing symptoms to converge due to spread of disease to similar brain areas. As a result of this convergence, studying end‐stage histopathological relationships may have also precluded detecting additional nuanced differences in amygdalar disease burden earlier in the disease course.

Our findings did not demonstrate significant associations between neuronal shrinkage or death and the abundance of Pick bodies in the amygdala. Rather, higher Pick body burden was associated with an abundance of large Pick cells, which remained despite their abnormal morphology. Pick body density was also positively associated with larger intact neurons. While this may suggest that larger amygdala neurons are more susceptible to Pick body formation, our findings suggest that perhaps early, diffuse tau accumulation in seemingly unaffected neurons accounts for these results. We have previously shown discordant relationships between inclusion and neuronal densities in FTLD due to tau and TDP‐43 [19, 46], suggesting that intraneuronal proteinopathy accumulation may not invariably lead to neuronal death. Additionally, we have shown that the dentate gyrus of the hippocampus retains its high neuronal density despite substantially greater Pick body inclusions than neocortical regions [20]. Like the hippocampus, prior studies have suggested that neurogenesis continues in the amygdala through adulthood, possibly through sustained repopulation from immature neurons in the paralaminar nucleus and adjacent corticoamygdaloid transition area [47, 48]. Therefore, it is possible that the amygdala and hippocampus show some resiliency to neuronal death in Pick disease as sites of continued neurogenesis.

A major strength of our study is its focus on a cohort of longitudinally well‐characterized bvFTD and PPA participants with autopsy confirmation of underlying 3R and 4R FTLD‐tauopathies, allowing us to characterize clinicopathologic relationships involving amygdalar vulnerability in greater detail than purely antemortem studies or postmortem studies that do not account for clinical diagnosis. Further, our histopathologic analyses, particularly in PiD cases, go beyond tau burden, providing insights into multifactorial biologic substrates of pathogenesis affecting the amygdala. Several limitations of our study are worth noting. First, our post‐mortem analysis utilized unilateral amygdala sections, limiting our ability to evaluate intra‐subject effects of hemispheric lateralization in our cohort, which is an important consideration given studies showing left lateralization of disease burden in PPA patients vs. bilateral or rightward involvement in bvFTD [19, 49, 50]. However, it is worth mentioning that the effect of hemisphere was not significant in any of our models. Second, while CBD and PSP cases frequently retained recognizable cytoarchitectonic landmarks within the basolateral amygdala, demarcations of subnuclei were not appreciable in PiD cases due to severe disease‐related atrophy and high pathologic burden. To ensure uniform uniformity of data acquisition and quantification, analyses were constrained to a whole‐region assessment of amygdala vulnerability rather than a fine‐grained assessment of specific amygdala subnuclei involved. Future high‐resolution MRI or stereological studies involving thicker, serial sections (e.g., fixed‐frozen, 40‐μm sections) will be necessary to map precise patterns of subnuclear vulnerability in this region. Third, while percent area occupied by HLA‐DR is a commonly used metric to measure activated microglia [51, 52], HLA‐DR immunopositivity also labels perivascular macrophages and does not exclusively distinguish activated from resting states. Future studies incorporating complementary markers (e.g., Iba1 and CD68) alongside stereologic counts of morphologically defined microglial phenotypes will help clarify associations between FTLD‐tau pathology and microglial response in the amygdala. Relatedly, while percent area occupied by AT‐8 provides an overview of phosphorylated tau burden, future studies should investigate isoform‐ and lesion‐type‐stratified tau lesions (e.g., using RD3 and ET3 staining). These approaches could provide insights into the molecular and cell‐type signatures contributing to differential amygdalar vulnerability between 3R and 4R FTLD‐tauopathies. Finally, while we identified statistically significant neuroinflammatory differences between clinical groups in our broader FTLD‐tau cohort (*N* = 47), analyses in the PiD group (*N* = 19) were constrained by a smaller sample size, and the neuroinflammatory associations observed should be interpreted cautiously. Likewise, replication in larger postmortem cohorts incorporating multi‐modal antemortem and postmortem markers is warranted.

In summary, results of this study highlight the important role of neuroinflammation in the relationship between tau accumulation and clinical phenotype in frontotemporal lobar degeneration. Furthermore, the size and abundance of Pick cells in our cohort suggest that neuronal alterations beyond shrinkage or death may contribute to clinical symptoms, as has been shown in prior studies of intrinsic amygdala activity and functional connectivity. This study contributes to our understanding of the complex relationship between pathology and clinical presentation in Pick disease, particularly in the amygdala, with broader implications for diagnosing and treating primary tauopathies. Although further translational validation is needed, assessing and targeting neuroinflammation in vulnerable regions implicated in behavioral and neuropsychiatric symptoms, such as the amygdala, may represent a possible intervention target for behavioral disruption and neuropsychiatric symptoms in frontotemporal lobar degeneration.

## AUTHOR CONTRIBUTIONS

R.K., C.G., and T.G. conceived and designed the study. R.K., A.K., and G.M. optimized the methodology. Q.M., R.J.C., and P.J. determined the appropriate neuropathologic diagnoses for the cases and prepared the postmortem samples for analysis. R.K. and N.G. analyzed the data. R.K., A.K., G.M., A.Z., A.M., C.N., and T.G. contributed to resource coordination and management and data curation. S.W., M.M.M., C.G., and T.G. provided supervision. R.K. drafted the initial manuscript and figures. All authors participated in the review and editing process and approved the final manuscript.

## FUNDING INFORMATION

This study was supported by grants from the National Institute on Aging (U01AG016976, P30AG013854, P30AG072977, R01AG062566, R01AG077444, R01AG056258, R56AG075600, K08AG065463, F31AG076318, T32AG020506), National Institute on Deafness and Other Communication Disorders (R01DC008552), National Institute of Neurological Disorders and Stroke (R01NS085770, R01NS075075, T32NS047987), the National Science Foundation's Graduate Research Fellowship (DGE‐1842165), and the Karen Toffler Charitable Trust. A total of 95 of this research was financed with federal funds. A total of 5 percent of this research project was financed with non‐governmental funds. This manuscript is the result of funding in whole or in part by the National Institutes of Health (NIH). It is subject to the NIH Public Access Policy. Through acceptance of this federal funding, NIH has been given a right to make this manuscript publicly available in PubMed Central upon the Official Date of Publication, as defined by NIH. The content is solely the responsibility of the authors and does not necessarily represent the official views of the National Institutes of Health.

## CONFLICT OF INTEREST STATEMENT

The authors report no competing interests.

## ETHICS STATEMENT

Written informed consent and agreement to enter the brain donation program were obtained from all participants in the study, and the study was approved by the Northwestern University Institutional Review Board and in accordance with the Helsinki Declaration.

## Supporting information


**Supplementary Figure 1.** 3R tau and 4R tau isoform specific staining. (A)–(D) In 2 bvFTD cases due to PiD (P7 and P9), inclusions in the right hemisphere hippocampal dentate gyrus granule cells were predominantly RD3+ for the 3R tau isoform, with minimal ET3+ for the 4R tau isoform. Neuropathological semi‐quantitative assessment indicated a moderate to severe level of neuronal tau inclusions, with glial tau inclusions ranging from mild to moderate. (E)–(H) Staining in 2 bvFTD cases due to CBD showed predominantly ET3+ (4R tau) inclusions and threads in the right hemisphere dentate gyrus granule cells (case P30) and the left putamen (case P26), with minimal RD3 positivity (3R tau). Semi‐quantitative neuropathological assessment showed a mild level of glial tau inclusions in the dentate (top) and severe level of glial tau inclusions in the putamen (bottom). (I)–(L) The left putamen in a case with PPA (P45) and a case with bvFTD (P40), both due to PSP, showed a similar pattern of predominant ET3 (4R tau) positivity. Neuropathological semi‐quantitative assessment indicated a severe level of glial tau inclusions in this region. Staining was completed as part of the neuropathological evaluation. All photomicrographs depicted were taken at 10× magnification.
**Supplementary Figure 2**. Convergence analysis of neuronal size. In 3 cases with PiD (bvFTD = P2 and P3; PPA = P17), analysis of neuronal size at increasing sampling fractions showed stable neuronal area means and standard deviations (both in μm^2^) at the 2.5% sampling fraction of the amygdala used for this study.

## Data Availability

The datasets used and/or analyzed during the current study are available from the corresponding author upon reasonable request (see: https://www.brain.northwestern.edu/scientists-students/collaborative-request.html).
